# Attitudes of Medical and Health Sciences Students towards Abortion in Jordan

**DOI:** 10.1155/2021/6624181

**Published:** 2021-04-26

**Authors:** Rami Saadeh, Mahmoud Alfaqih, Asma Odat, Mohammed Z. Allouh

**Affiliations:** ^1^Department of Public Health and Community Medicine, Faculty of Medicine, Jordan University of Science and Technology, Irbid, Jordan; ^2^Department of Physiology and Biochemistry, Faculty of Medicine, Jordan University of Science and Technology, Irbid, Jordan; ^3^Faculty of Pharmacy, Jordan University of Science and Technology, Irbid, Jordan; ^4^Department of Anatomy, College of Medicine and Health Sciences, United Arab Emirates University, Al Ain, UAE

## Abstract

**Background:**

Jordan laws on permitting abortion are considered moderate. Religion is one of the key determinants of people's attitudes towards abortion and plays a crucial role in people's readiness to accept or refute this practice. In this study, we examined the attitudes of medical and health sciences students towards abortion.

**Methods:**

In this cross-sectional study, a self-administered questionnaire survey was distributed to students at Jordan University of Science and Technology. Attitudes towards abortion were tested using 16 items that were included in the survey. Descriptive statistics and logistic regression models were used in the analysis.

**Results:**

A total of 1324 students in the medicine and dentistry colleges participated in the study. Two-thirds of the participants were women. Most participants were 20–25 years old, and they grew up in a family of 6–8 members. The overall attitude towards abortion was negative, except if the pregnancy was a threat to the mother's life (91.5%) or if the conception occurred from rape (54.2%). Otherwise, the students indicated that every conceived child has the right to be born (76.8%) and that abortion is considered murder (53.1%). Furthermore, the students who were more likely to support abortion were those attending the medical college, living in a city, and/or raised in smaller families (*p* = 0.04).

**Conclusions:**

Compared with other students, medical students were more supportive of abortion. This implied the necessity to include training on safe abortion in the medical curriculum and increase public awareness of the importance of safe abortion.

## 1. Introduction

Abortion refers to the termination of the conception of a fetus in the womb of a pregnant woman by removing fetal tissues, fetal membranes, and the placenta [1]. Historically, it is an old procedure that is judicially and culturally accepted in many regions worldwide [2]. However, the perception towards abortion might vary among different regions or subclasses of the same region. The reasons behind this variation are very complex and might be explained by the fact that perception towards abortion is influenced by many factors. For example, several cultures and religions around the world prohibit abortion altogether or draw restrictions on its use, and women who live in these situations might become victims of social stigmatism or explicit physical harm [3]. Consequently, pregnant women under these circumstances were more likely to seek unsafe abortions to terminate their pregnancies. Religions and cultures that prohibit abortion or limit its use view abortion as a deliberate termination of a vital and sacred life [4, 5]. Comparatively, some researchers claim that abortion could be safe and beneficial to women [6, 7]. Women right activist groups view abortion as a decision to be made solely by the pregnant mother without any external or background influences [8].

Countries in the Middle East and North Africa (MENA) region, including Jordan, are considered conservative countries regardless of the constant changes in the cultural aspects among young individuals. The religious rooting and strict traditions impede the complete acceptance of abortion and its conflicting issues that play essential roles in the legalization and formulation of laws on abortion [5, 9]. Based on most studies from sub-Saharan Africa and Southeast Asian countries, religion was identified as the most important factor that influenced the attitudes of healthcare providers towards induced abortions [3]. Islamically, the soul or the spirit of the fetus is mentioned in the Quran, which is the holy book of Muslims, and is named “Al Ruh.” There is a consensus among Muslim scholars that ensoulment happens at the age of four months in the womb, which is considered when the fetus's life begins. Thus, abortion after ensoulment is prohibited except in exceptional circumstances [10], which are juridically authorized by Muslim scholars but not biblically mentioned in the Quran [11]. Therefore, abortion after ensoulment, without reason allowed by Islamic law, is considered murder [10]. Nonetheless, there is a variety of opinions regarding the permissibility of abortion among the different Islamic schools [5, 12]. Jordan is a Muslim majority, with an estimated 92% population identifying themselves as Sunni Muslims [13]. Abortion in Jordan is based on French colonial law, which allows abortion to save the life or preserve the health of a woman. In 1971, a public health law in Jordan allowed abortion for mental health reasons [5]. However, like other countries in the region, Jordan's culture is changing, and new behaviors have frequently been emerging, making people's opinions on such issues unpredictable [14].

To explore the attitude of the Jordanian community on abortion, it is imperative to shed light on a critical portion of it, which is the students' knowledge and attitude towards abortion. This would facilitate the planning of training programs that can provide the required level of knowledge and skills to deal with this medical and social issue. In addition, to our best knowledge, there has been no data in the region on the attitude of students of medical and health-related sciences towards abortion. Therefore, this study is aimed at examining the knowledge and attitude of medical and health sciences students towards abortion.

## 2. Methods

### 2.1. Design and Setting

This was a cross-sectional study performed through a survey on students of health and medical sciences at Jordan University of Science and Technology (JUST), a public university located in Northern Jordan. JUST is one of the ten public universities in Jordan and provides only science majors, including health-related majors, such as medicine, dentistry, nursing, pharmacy, and allied medical sciences, in addition to all major branches of engineering. The total number of students at JUST is approximately 25,000. The study was approved by the institutional review board committee at JUST (#75-2019). The committee waived the need for written or verbal consent since the study was based on a questionnaire filled voluntarily. The questionnaire assessed the attitude of students towards abortion.

### 2.2. Instrument

The questions in the survey were based on a questionnaire developed by Sloan [15]. However, for this study, the survey was modified to reflect some cultural aspects that might affect the views on abortions. The survey questions were reviewed by 12 experts, including physicians and public health professionals. The survey was modified based on the recommendations by the experts who believed that the instrument appeared reasonable. A major modification was the change to a three-scale questionnaire, which comprised the following three choices: agree, do not agree, and neutral. Sloan used a 6-level scale, and the reduction to a 3-level scale was considered more suitable to reflect the students' opinion and provide a sharper image of their attitude towards abortion. Furthermore, questions that were found similar were combined in a single question to avoid repetition. The questionnaire was originally developed in English and then translated into Arabic. An academic expert, blinded to the original English survey, had translated the Arabic version into English to verify that the original meanings of the questions are preserved. After validating language proficiency, the translated Arabic version was pilot tested among nine experts who initially validated the survey and ten students. The modifications made based on the feedbacks included changes in two questions. The survey contained eight questions that were related to demographic information (i.e., age, sex, past and current number of family members, major of education, level of education, years of completion of current education, and place of residence) and 16 questions that were related to the students' attitude towards abortion. The survey was prepared on Google forms, which is an online survey instrument.

### 2.3. Sampling

A link to the survey, which was developed through Google forms, was distributed in May 2019 to the students of JUST through Facebook. In Jordanian universities, most students share and distribute information through Facebook groups. However, the number of students who had Facebook accounts or who routinely used their Facebook accounts for these groups was unknown. Despite this limitation, this data collection method was more convenient for students and researchers, rapid and efficient, and usually resulted in a high response rate [16]. A class representative from each major in the university was contacted to assist with data collection from his/her classmates. A short meeting with each of the class representatives was conducted to explain the distribution of the link through the Facebook groups of the students. To unify the data collection method, each class representative was asked to identify the five most common active Facebook groups in his or her college and post the link to these groups once a week for four weeks. The link included a preface that explained the purpose of the study; the inclusion criterion, which was any student who was currently registered in JUST; and the consent form, which included a statement that ensured optionality, anonymity, and confidentiality of the study and the right to withdraw anytime.

### 2.4. Statistical Analysis

Descriptive statistics were used to describe the demographic characteristics and the knowledge and attitude on abortion. Multinomial logistic regression was used to examine the relationship of the demographic characteristics of the students with their views on the following: (1) if abortion is considered murder, (2) if abortion is permissible for pregnancies of unmarried adolescents, (3) if laws and regulations will support abortion in the future, (4) if talking about abortion with patients is considered a social flaw, and (5) if abortion should be permitted in cases of congenital fetal anomaly. Those who “do not agree: were coded as “0,” “neutral” as “1,” and “agree” as “2.” However, a comparison of a statistical change in probability was made between those who “do not agree” and those who “agree” because “neutral” is not considered part of any of the two options and cannot be eliminated. Analyses were performed using the Statistical Package for the Social Sciences “SPSS” software version 23. The level of significance for all statistical tests was set at *p* < 0.05.

## 3. Results

There were 1324 students who completed the survey. Approximately, two-thirds of the participants were females. Most of the participants were in the age range of 20–25 years. The majority of participating students belonged to either the college of medicine or the college of dentistry, and most of them grew up in a family of 6–8 members (Table 1). Most of the students who completed the survey were undergraduates (95%). Furthermore, the ratio of students who lived in a city to those who lived in a village was 3 : 1. The responses of the students on their views about abortion are shown in Table 2. In general, the students were not supportive of abortion unless it was a threat to the mother's life or if a woman conceived the fetus from rape. More than half of the students did not consider abortion as an excuse if an infant was detected to have Down's syndrome. Although most students considered the fetus a human from the moment of conception (60%) and that every conceived fetus has the right to be born (76.8%), many agreed that abortion is permissible before the soul is breathed into the fetus, not after (45.7%).

On multinomial regression relationships, we found that being a student in the college of medicine, living in a city, and having been raised in smaller families were associated with more positive or supportive views towards abortion. For instance, compared with students from other colleges, those from the college of medicine were less likely to view abortion as murder (OR 0.339, 95% CI 0.182–0.632, *p* < 0.01), more likely to support the abortion of pregnancies of unmarried adolescents (OR 2.591, 95% CI 1.516–4.430, and *p* < 0.01), and were the only students to expect that laws and regulations should support abortion in the future (OR 1.808, 95% CI 1.109–2.947, and *p* = 0.017).

Students who were raised in families of 3–5 people were less likely to view abortion as murder (OR 0.319, 95% CI 0.179–0.572, and *p* < 0.01) and more likely to expect that laws will support abortion in the future (OR 1.886, 95% CI 1.079–3.014, and *p* < 0.01). Moreover, they were more likely to support the abortion of pregnancies of unmarried adolescents (OR 2.865, 95% CI 1.701–4.826, and *p* < 0.01). A similar trend was observed between students raised in families of 6–8 people (OR 1.735, 95% CI 1.065–2.825, and *p* = 0.02) and those raised in families of 9 or more people.

Compared to students who lived in villages, those who lived in cities were less likely to view abortion as murder (OR 0.623, 95% CI 0.412–0.936, and *p* < 0.01) and were more likely to support the abortion of pregnancies of unmarried adolescents (OR 1.683, 95% CI 1.196–2.370, and *p* < 0.01). Talking about abortion with patients was considered a social flaw by more students who were less than 25 years old (<20 years: OR 0.300, 95% CI 0.132–0.681, and *p* < 0.01 and 20–25 years: OR 0.433, 95% CI 0.207–0.908, and *p* = 0.02) and by more undergraduate students than graduate students (OR 0.401, 95% CI 0.189–0.848, and *p* = 0.01). Compared to female students, male students were more likely to consider discussions about abortion with patients as a social flaw (OR 2.466, 95% CI 1.615–3.676, and *p* < 0.01) and were less likely to agree that abortion should be permitted if the fetus has congenital anomalies (OR 0.712, 95% CI 0.418–0.961, and *p* = 0.02). Furthermore, students who were in the first year of their education at the university were more likely to view abortion as murder (OR 1.721, 95% CI 1.186–2.503, and *p* < 0.01) and were less likely to agree that abortion should happen if the fetus has congenital abnormalities (OR 0.578, 95% CI 0.401–0.811, and *p* < 0.01).

## 4. Discussion

The legalization of abortion cannot be determined without considering the complexity of the issues entailed within abortion. There are several reasons that make women choose abortion; some of these include forced marriage, adolescent marriage, unwanted pregnancies, low socioeconomic status, sexual violence, and intimate relationship of unmarried couples that end up in pregnancy. These factors could implicate an emotional, social, or economic burden that makes pregnant women choose abortion [3, 17].

Students in this study varied in their views on abortion. However, most students demonstrated a conservative attitude towards abortion. In this study and other studies, abortion without a considerable reason has been considered murder [18, 19]. Medical interns from India reported that unmarried women should not abort without their partner's consent [19]. On the other hand, a South African study showed that 80% of students believed that abortion should be allowed for any reason [20]. In addition, 62% of medical students in the United Kingdom showed a general proabortion attitude [21]. In the present study, the only two reasons viewed by the students as an excuse for abortion were if the mother was threatened by her pregnancy or if the mother conceived a fetus from rape. Our findings were consistent with the results of a study conducted from 1983 to 1990 on medical students at The Johns Hopkins University School of Medicine, where the students agreed that abortion should be permitted only if the life of the mother was threatened or if the conception resulted from rape [22]. However, the percentage of those who supported abortion for conceptions from rape was much higher in the Johns Hopkins study than in this study (94% vs. 54%). Moreover, 46% of the fourth-year medical students in the Johns Hopkins study believed that abortion should be provided on demand, whereas 53% of the JUST students in this present study considered abortion to be murder. Given such differences, a negative view towards abortion was significantly more frequent in the JUST students than in the students at Johns Hopkins. Other studies also supported abortion for such reasons (i.e., rape and threat to mother's life) [23, 24].

In this study, the students considered abortion as murder of an unborn infant. However, they thought that the threat to the mother's life by pregnancy was even worse. They agreed with the views of Jordanian law and the religious regulations, which allow abortion to save the life or preserve the health of a woman. Moreover, the students' views were like the religious opinion of most Muslim scholars who permitted abortion of a pregnancy that resulted from rape, especially during war situations. However, some Muslim scholars have regulated the permission to abort in rape cases to the first trimester [5, 8, 12, 25].

Countries vary in their policies and laws on abortion, as shown by the Center of Reproductive Life, which classifies countries into five categories according to the degree of permitting abortion [2]. Based on this categorization, most countries in Africa and South America are very conservative and have laws that do not allow abortion under any circumstances, whereas other countries in Europe and North America allow abortion upon request. Jordan falls in category 3, which allows abortion to preserve health. Jordan and most MENA region countries make their laws mainly in accordance with the religious laws. In Jordan, approximately 92% of the population is Muslim, and 6–7% is Christian. Both religions are not supportive of abortion, unlike some other religions with more lenient views [18]. It seemed that the religious opinions of Islam and Christianity might have affected the students' attitude towards abortion. This was reflected by the students' views (45.7%) on permitting abortion before gaining the soul (ensoulment). From an Islamic perspective, the ensoulment or the breathing of the soul into the fetus occurs after 120 days, which is the evidence used by many Islamic scholars that abortion should not be allowed after that [4, 5, 11, 12].

The medical students were more likely to support abortion than the students of other disciplines. This finding was in line with the findings of studies from the United States, South Africa, Brazil, and Malaysia, where medical students were more likely to support the provision of abortion and were more open to receive education on abortion than other students [20, 26–28]. The medical education and clinical training convinced the medical students that abortion might be unavoidable [29, 30]. Additionally, the attitude towards abortion was significantly influenced by the year of the study as the first-year students had more negative opinions on abortion than the upper-level students. This was comparable with a study from the United Kingdom where 70% of second-year medical students had a prochoice opinion on abortion compared to 54% of first-year students [21].

Although the man-to-woman ratio in this study was 1 : 2, the large sample size was still considerable for comparison. The male students in this study had more conservative views on abortion than female students. In 2015, a review on abortion revealed that the attitude towards abortion was more conservative among women than among men [3]. However, in the reviewed studies, the participants were nurses and healthcare providers in Asian and South Asian countries and had a significantly higher level of knowledge and experience than those in our study population. Moreover, the cultural variations may have contributed to the sex-related differences in attitude towards abortion between our study and the reported South Asian studies.

If the general attitude of the students in this study reflects on the public opinion in the community about abortion, it could possibly imply a hindrance to apply proper health policy planning and implementation. In the long term, if this issue is not addressed among health practitioners, policymakers, and the public, an increase in the overall maternal mortality and morbidity rates from unsafe abortions is expected since unsafe abortions are relatively common in societies that hold negative opinions on abortion [11, 31]. Notably, laws that regulate abortion may be protective of women's rights in certain countries; however, the public, especially people in the low socioeconomic classes, may be unaware of these laws and regulations [24]. Under these circumstances, the implementation of awareness campaigns to better educate the public on the laws regulating pregnancy termination and the risks associated with unsafe abortions becomes mandatory [32]. These campaigns may be directed towards medical and health practitioners who are in direct contact with pregnant women, able to detect unsafe abortions, and capable of educating women on the laws and regulations that govern abortion to correct any misconceptions on this topic [33].

### 4.1. Limitations

A major limitation of this study was that the study relied on Facebook for data collection. Therefore, individuals who did not have Facebook accounts and internet access on a computer or phone were excluded [16]. This could be considered a possible source of bias in sampling. Another limitation was that the number of students who came across the link but did not participate in the survey and the students who did not complete the survey was unknown. Therefore, the response rate could not be recognized.

## 5. Conclusions

This study is unique because of the scarcity of studies that discuss this issue in the MENA region. The students from different health disciplines had a conservative view towards abortion, which was possibly related to their religious beliefs. However, the supportive views of medical students implied that this group, specifically, might be willing to provide abortion. Consequently, political and academic commitments would be required to introduce the concept of abortion in the medical curriculum; develop training programs to enable and guide medical students to practice safe abortion when needed; provide resources for a safe practice of abortion; and create a positive environment on the practice of safe abortion through comprehensive educational and awareness programs for the public, especially the younger generations. Such interventions could reduce the negative attitude towards abortion and secure safe and protected abortion for women who seek it.

## Figures and Tables

**Table 1:** Demographic information of participants.

Characteristics	Number (%)
*Sex*	
Male	430 (32.5%)
Female	890 (67.2%)
Missing	4 (0.3%)
Total	1324 (100%)
*Age*	
Less than 20 years old	416 (31.4%)
20–25 years old	835 (63.1%)
Older than 25 years old	71 (5.4%)
Missing	2 (0.2%)
Total	1324 (100%)
*Size of the family when you grew up*	
3–5 people	346 (26.1%)
6–8 people	821 (62.0%)
9 people or more	151 (11.4%)
Missing	6 (.5%)
Total	1324 (100%)
*Size of your current family (if you are married)*	
3–5 people	115 (57.5%)
6–8 people	80 (40%)
9 people or more	5 (2.5%)
Total	200 (100%)
*Place of residence*	
City	1019 (77.0%)
Village	301 (22.7%)
Missing	4 (.3%)
Total	1324 (100%)
*Education level*	
Bachelor	1243 (93.9%)
Master	73 (5.5%)
Missing	8 (.6%)
Total	1324 (100%)
*Your college of current education*	
College of medicine	584 (44.1%)
College of dentistry	297 (22.4%)
College of nursing	19 (1.4%)
College of pharmacy	140 (10.6%)
College of applied medical sciences	160 (12.1%)
Other colleges	122 (9.2%)
Missing	2 (.2%)
Total	1324 (100%)
*Years of completion of current education*	
2 years or less of education	576 (43.4%)
2–4 years of education	390 (29.5%)
4 years or more of education	348 (26.3%)
Missing	10 (.8%)
Total	1324 (100%)

Distribution of demographic characteristics is explained by numbers and percentages.

**Table 2:** Attitude towards abortion.

Attitude towards abortion	Number (%)
*Do you think that abortion is lawful in Jordan?*	
No	972 (73.4%)
Yes	349 (26.4%)
Missing	3 (.2%)
Total	1324 (100%)
*Abortion is an accepted way to solve unwanted pregnancy*	
I do not agree	831 (62.8%)
Neutral	305 (23.0%)
I agree	186 (14.0%)
Missing	2 (0.2%)
Total	1324 (100%)
*A mother should feel obligated to bear a child she has conceived*	
I do not agree	154 (11.6%)
Neutral	261 (19.7%)
I agree	907 (68.5%)
Missing	2 (.2%)
Total	1324 (100%)
*Every conceived child has the right to be born*	
I do not agree	105 (7.9%)
Neutral	200 (15.1%)
I agree	1017 (76.8%)
Missing	2 (.2%)
Total	1324 (100%)
*Abortion is permissible if pregnancy is a threat to a mother's life*	
I do not agree	19 (1.4%)
Neutral	91 (6.9%)
I agree	1212 (91.5%)
Missing	2 (.2%)
Total	1324 (100%)
*Abortion should occur if the fetus has congenital anomalies or genetic diseases*	
I do not agree	343 (25.9%)
Neutral	462 (34.9%)
I agree	514 (38.8%)
Missing	5 (.4%)
Total	1324 (100%)
*Abortion should occur if the fetus has Down syndrome*	
I do not agree	758 (57.3%)
Neutral	354 (26.7%)
I agree	208 (15.7%)
Missing	4 (.3%)
Total	1324 (100%)
*Abortion must be considered murder*	
I do not agree	234 (17.7%)
Neutral	380 (28.7%)
I agree	703 (53.1%)
Missing	7 (.5%)
Total	1324 (100%)
*People should not look inferior to women who choose to have an abortion*	
I do not agree	208 (15.7%)
Neutral	509 (38.4%)
I agree	604 (45.6%)
Missing	3 (.2%)
Total	1324 (100%)
*Abortion should be an option available to unmarried pregnant adolescents*	
I do not agree	691 (52.2%)
Neutral	281 (21.2%)
I agree	349 (26.4%)
Missing	3 (.2%)
Total	1324 (100%)
*Abortion should be available to women who have conceived from rape*	
I do not agree	252 (19.0%)
Neutral	352 (26.6%)
I agree	717 (54.2%)
Missing	3 (.2%)
Total	1324 (100%)
*The fetus must be considered human from the moment of conception*	
I do not agree	256 (19.3%)
Neutral	272 (20.5%)
I agree	794 (60.0%)
Missing	2 (.2%)
Total	1324 (100%)
*Abortion is permissible before the soul is breathed into the fetus and not after it*	
I do not agree	236 (17.8%)
Neutral	474 (35.8%)
I agree	605 (45.7%)
Missing	9 (.7%)
Total	1324 (100%)
*Abortion must be prevented because:*	
I am not against abortion	303 (22.9%)
Against religion	437 (33.0%)
Against traditions and norms	1 (.1%)
Murder	575 (43.4%)
Missing	8 (.6%)
Total	1324 (100%)
*Discussion with patients about abortion is a social flaw*	
I do not agree	884 (66.8%)
Neutral	330 (24.9%)
I agree	102 (7.7%)
Missing	8 (.6%)
Total	1324 (100%)
*Laws and legislation will support abortion in the future because of changes in the culture of the society*	
I do not agree	426 (32.2%)
Neutral	407 (30.7%)
I agree	482 (36.4%)
Missing	9 (.7%)
Total	1324 (100%)

Students' responses towards items regarding abortion are represented as numbers and percentages.

## Data Availability

All data generated or analyzed during this study are included in this published article.
